# The complete chloroplast genome sequence of Korean *Lonicera japonica* and intra-species diversity

**DOI:** 10.1080/23802359.2018.1502637

**Published:** 2018-08-23

**Authors:** Shin-Jae Kang, Hyun-Seung Park, Hyun Jo Koo, Jee Young Park, Dong Young Lee, Kyo Bin Kang, Sang Il Han, Sang Hyun Sung, Tae-Jin Yang

**Affiliations:** a Department of Plant Science, Plant Genomics and Breeding Institute, Research Institute of Agriculture and Life Sciences, College of Agriculture and Life Sciences, Seoul National University, Seoul, Republic of Korea;; b College of Pharmacy and Research Institute of Pharmaceutical Science, Seoul National University, Seoul, Republic of Korea;; c Medicinal Plant Garden, College of Pharmacy, Seoul National University, Koyang, Republic of Korea

**Keywords:** *Lonicera japonica*, chloroplast genome, genome sequence, golden-and-silver honeysuckle, Japanese honeysuckle

## Abstract

*Lonicera japonica* is a traditional medicinal plant well known for its anti-inflammatory effect. The complete chloroplast genome sequence of *L. japonica* collected from Korea was obtained by *de novo* assembly using whole genome sequence data. The chloroplast genome is 155,060 bp in length, containing 88,853 bp in a large single copy (LSC), 18,653 bp in a small single copy (SSC) and 23,777 bp in a pair of inverted repeats (IRs). A total of 112 genes including 78 protein-coding genes and 34 structural RNA genes were identified. The sequence comparison of two *L. japonica* collected from Korea and China revealed 48 single nucleotide polymorphisms (SNPs) and 45 insertions/deletions (InDels). In addition, phylogenetic analysis represented intraspecific diversity within *L. japonica* species collected in Korea and China.

The *Lonicera* genus belongs to the Caprifoliaceae family which is close to the Rubiaceae family and consists of approximately 200 species that are mainly distributed in East Asia. Among them, about 100 inhabit in China, about 25 in Japan and about 30 in Korea. Many *Lonicera* species have been used as herbal medicines. *L. japonica*, called golden-and-silver honeysuckle or Japanese honeysuckle, has been widely used in traditional herbal medicine (Peng et al. 2000), and its flower bud also has been prescribed to treat some infectious diseases due to its anti-inflammatory and antiviral effects (Chang and Hsu 1992). These effects come from many active compounds identified in the stems and leaves of *L. japonica* (Shang et al. 2011). Previously, a chloroplast genome sequence of *L. japonica* collected from China (China collection) has been reported (He et al. 2017). In this study, we characterized the complete chloroplast genome sequence of *L. japonica* collected from Korea (Korea collection) and compared it with the Chinese *L. japonica* chloroplast genome.

The plant sample was collected from Medicinal Plant Garden, College of Pharmacy, Seoul National University, Koyang, Korea. Total genomic DNA was extracted from fresh leaves using a modified cetyltrimethylammonium bromide protocol (Allen et al. 2006) and sequenced by the Illumina MiSeq platform (Illumina, San Diego, CA). Whole genome sequence data of 4.1 Gb in paired-end reads were obtained and assembled *de novo* using CLC genome assembler (v. beta 4.6, CLC Inc., Aarhus, Denmark) as previously described (Kim et al. 2015a). The gene annotation of the complete chloroplast genome was performed using GeSeq (https://chlorobox.mpimp-golm.mpg.de/geseq.html), followed by manual confirmation using Artemis programme (Rutherford et al. 2000) and BLAST searches.

Complete chloroplast genome of *L. japonica* (Korea collection) was 155,060 bp in length, and separated into four distinct regions: a large single copy (LSC) (88,853 bp), a small single copy (SSC) (18,653 bp), and a pair of inverted repeats (IRs) (each 23,777 bp). In Korea *J. japonica* collection chloroplast genome, a total of 112 genes including 78 protein-coding genes, 30 tRNA genes, and 4 rRNA genes were identified.

Chloroplast genome comparison between Korea and China *L.* japonica collections revealed that the Korea collection was 18 bp shorter than China collection, 5 bp and 19 bp shorter in LSC and SSC regions, respectively, but 3 bp longer in each IR region. We identified 48 SNPs and 45 InDels between these two *L. japonica* collections that show richer intra-species diversity such as *Panax ginseng* and *Brassica* species (Kim et al. 2015b, 2018; Joh et al. 2017; Nguyen et al. 2017, 2018).

A phylogenetic tree based on complete chloroplast genome sequences was constructed with seven other species including two in Caprifoliaceae and five in Adoxaceae (Figure 1). As expected, Korea and China collections of *L. japonica* were clustered together and grouped with other Caprifoliaceae species. Based on the branch lengths, *L. japonica* (China collection) has a long branch and it is thought more diversified from their common ancestor than *L. japonica* (Korea collection).

**Figure 1.**
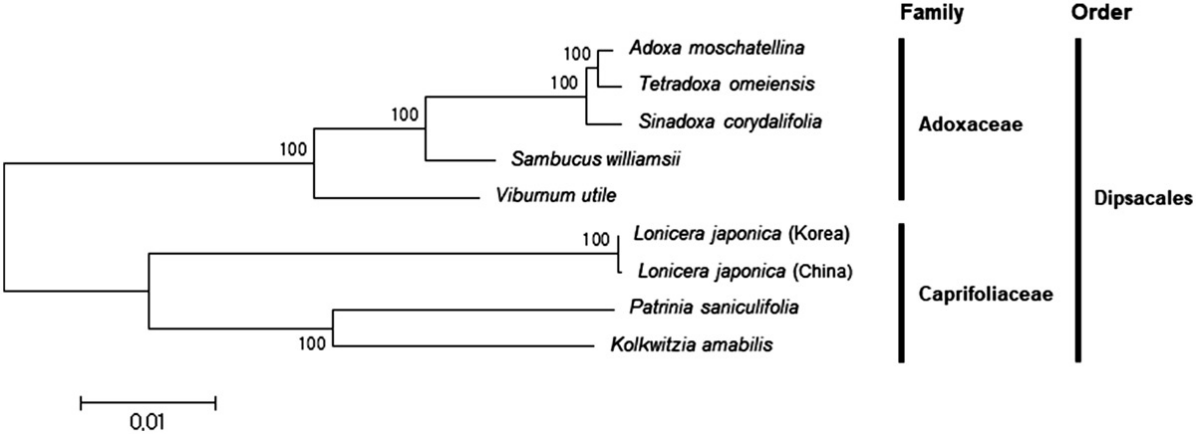
Phylogenetic tree of *Lonicera* and other related species in Dipsacales. The tree was constructed using complete chloroplast genome sequences of the nine species and analysed by the neighbour-joining method with 1000 bootstrap values in MEGA 6.0 (Tamura et al. 2013). The numbers in the nodes indicate bootstrap support values. The chloroplast genome sequences used for this tree are: *Adoxa moschatellina*, NC_034792.1; *Kolkwitzia amabili*, NC_029874.1; *Lonicera japonica* (Korea), MH028738; *Lonicera japonica* (China), NC_026839.1; *Patrinia saniculifolia*, NC_036835.1; *Sambucus williamsii*, NC_033878.1; *Sinadoxa corydalifolia*, NC_032040.1; *Tetradoxa omeiensis*, NC_034793.1; *Viburnum utile*, NC_032296.1.
